# Supplementary material for: The amoeboid migration of monocytes in confining channels requires the local remodeling of the cortical actin cytoskeleton by cofilin-1
Source: Sci Rep. 2024 May 3;14:10241. doi: 10.1038/s41598-024-60971-1 (PMC11068741; doi:10.1038/s41598-024-60971-1)
Supplement: Supplementary file 1 — Supplementary Legends. [file 41598_2024_60971_MOESM1_ESM.pdf]

## Movies

**Movie 1.** Motile THP-1 monocyte-like cell with LifeAct-EGFP. Microchannels are coated with VCAM-1 (1 µg/mL) and are 3 µm in height, 8 µm in width, and 100 µm in length.

**Movie 2.** Motile THP-1 monocyte-like cell with RLC-EGFP. Microchannels are coated with VCAM-1 (1 µg/mL) and are 3 µm in height, 8 µm in width, and 100 µm in length.

**Movie 3.** Non-motile THP-1 monocyte-like cell with LifeAct-EGFP. Microchannels are coated with VCAM-1 (1 µg/mL) and are 3 µm in height, 8 µm in width, and 100 µm in length.

**Movie 4.** Central focal plane of a non-motile THP-1 monocyte-like cell with LifeAct-EGFP treated with a cofilin-1 siRNA. Microchannels are coated with VCAM-1 (1 µg/mL) and are 3 µm in height, 8 µm in width, and 100 µm in length.

**Movie 5.** Ventral focal plane of a non-motile THP-1 monocyte-like cell with LifeAct-EGFP treated with a cofilin-1 siRNA. Microchannels are coated with VCAM-1 (1 µg/mL) and are 3 µm in height, 8 µm in width, and 100 µm in length.

**Movie 6.** Pseudo colored movie of cofilin-1-EGFP in a motile THP-1 monocyte-like cell. Microchannels are coated with VCAM-1 (1 µg/mL) and are 3 µm in height, 8 µm in width, and 100 µm in length.
